# The Evolution of Phage Therapy: A Comprehensive Review of Current Applications and Future Innovations

**DOI:** 10.7759/cureus.70414

**Published:** 2024-09-28

**Authors:** Kaushik Sahoo, Supriya Meshram

**Affiliations:** 1 Microbiology, Jawaharlal Nehru Medical College, Datta Meghe Institute of Higher Education and Research, Wardha, IND

**Keywords:** antibiotic resistance, bacteriophages, clinical applications, genetic engineering, innovations, phage therapy

## Abstract

Phage therapy, using bacteriophages to target and destroy bacteria, has evolved significantly from its early 20th-century inception to its modern resurgence as a promising alternative to antibiotics. This review explores the historical development of phage therapy, detailing its initial successes, subsequent decline with the rise of antibiotics, and recent revival in response to increasing antibiotic resistance. We examine the fundamental mechanisms of phage therapy, including the specificity of phages for bacterial targets and their ability to combat resistant strains. Current applications are discussed, highlighting the use of phage therapy in treating chronic infections, personalizing treatment strategies, and its role in veterinary and food safety contexts. Innovations in phage therapy, driven by advancements in genetic engineering and synthetic biology, are also reviewed, showcasing the development of engineered phages, phage libraries, and novel delivery systems. Despite its potential, phage therapy faces challenges such as regulatory hurdles, safety concerns, and issues related to bacterial resistance to phages. The review underscores the need for ongoing research and clinical trials to address these challenges and to integrate phage therapy into modern treatment paradigms. By offering a detailed overview of the evolution, current status, and future directions of phage therapy, this review aims to highlight its potential as a crucial component in the fight against antibiotic-resistant infections and to inform future research efforts.

## Introduction and background

Phage therapy is a novel treatment approach that employs bacteriophages, viruses that specifically infect and destroy bacteria, to combat bacterial infections [1]. Unlike traditional antibiotics that indiscriminately target a wide range of bacteria, phages are highly specific to their bacterial hosts. This specificity enables phage therapy to selectively target pathogenic bacteria while sparing the beneficial microbiota, potentially reducing collateral damage [2]. The therapeutic use of phages capitalizes on the natural predator-prey relationship between these viruses and bacteria, providing a targeted strategy for managing infections that resist conventional treatments [3].

The concept of phage therapy emerged in the early 20th century with the discovery of bacteriophages by Félix d’Hérelle in 1917. d’Hérelle's observation that these viruses could effectively lyse bacteria in infected patients marked a pivotal moment in medical microbiology [4]. Following this discovery, phage therapy saw widespread use, particularly in Eastern Europe and the Soviet Union, where it was utilized to treat various bacterial infections. However, the development and widespread adoption of antibiotics in the 1940s led to a decline in the use of phage therapy, overshadowed by the broad-spectrum efficacy of antibiotics. Despite this decline, the rise of antibiotic-resistant bacterial strains in recent years has rekindled interest in phage therapy as a viable alternative or adjunct to conventional treatments [2].

The escalation of antibiotic resistance has become a critical global health issue, complicating the treatment of common bacterial infections and contributing to increased rates of morbidity and mortality [5]. The overuse and misuse of antibiotics have accelerated the emergence of resistant bacterial strains, diminishing the effectiveness of many of our most powerful antibiotics. This growing crisis underscores the urgent need for alternative therapeutic strategies, highlighting the potential of phage therapy to address infections that no longer respond to traditional antibiotics [5].

Phage therapy presents a promising solution to the problem of antibiotic resistance. Its ability to specifically target resistant bacteria while preserving the host's normal microbiota offers a distinct advantage over broad-spectrum antibiotics [6]. Furthermore, recent advancements in genetic engineering and synthetic biology are enhancing the efficacy and versatility of phage therapy, expanding its potential applications across a range of bacterial infections. This review aims to comprehensively examine the evolution of phage therapy, exploring its historical development, current applications, and future innovations. By delving into the mechanisms of action, recent technological advancements, and ongoing challenges, this review seeks to elucidate the role of phage therapy in modern medicine and its potential to transform the treatment of bacterial infections in the face of rising antibiotic resistance [7].

## Review

Historical context

The historical context of phage therapy illustrates a compelling trajectory of discovery, initial success, decline, and recent resurgence [8]. Bacteriophages, or phages, were first identified independently by Frederick William Twort in 1915 and Félix d'Hérelle in 1917 [9]. d'Hérelle's contributions were particularly notable, as he demonstrated the potential of phages to combat bacterial infections. By isolating phages from the feces of dysentery patients, d'Hérelle revealed their antibacterial properties and proposed their use as both prophylactic and therapeutic agents. This pioneering work led to early phage therapy trials for diseases such as typhoid fever and cholera in the 1920s, establishing a foundation for exploring phage therapy as an innovative approach to treating bacterial infections [9]. In the early 20th century, phage therapy gained significant traction, especially in Eastern Europe and the Soviet Union, where it was extensively researched and applied [2]. In the United States, phage therapy was first used therapeutically in 1922, with several pharmaceutical companies beginning to produce phage preparations for treating infections. Initial reports of success generated enthusiasm for phage therapy, and numerous studies documented its effectiveness against various bacterial infections. The approach was considered a promising alternative to existing treatments, leading to its broad application across different bacterial diseases [9]. The decline of phage therapy began in the 1940s with the advent of antibiotics, particularly penicillin, which provided broad-spectrum antibacterial activity and became the standard treatment for bacterial infections [8]. This decline was further exacerbated by issues related to the scientific rigor of early phage therapy studies, which often lacked proper controls and were published in non-English journals, limiting their accessibility to Western scientists. Additionally, the specific nature of phages meant that many preparations were ineffective against certain bacterial strains, undermining confidence in their therapeutic potential [8]. Despite this decline, interest in phage therapy has experienced a resurgence in recent years due to the growing issue of antibiotic resistance. Researchers are revisiting phage therapy as a viable treatment option, bolstered by advancements in our understanding of phage biology and the development of more rigorous clinical trials [2]. Phage therapy continues to be practiced in countries such as Georgia and Poland, where established centers actively explore its therapeutic applications. This revival underscores a renewed recognition of phage therapy's potential as an alternative treatment in the face of rising antibiotic resistance, marking a new chapter in its historical evolution [2].

Mechanism of action

The mechanism of action of phage therapy involves a complex interplay between the life cycle of bacteriophages, their interactions with bacterial targets, and the host immune response. Understanding these mechanisms is essential for effectively applying phage therapy in clinical settings [6]. Bacteriophages can follow two primary life cycles: the lytic and lysogenic cycles. In the lytic cycle, phages attach to a susceptible bacterial host, inject their genetic material, and hijack the bacterial machinery to replicate themselves [10,11]. This process leads to the lysis (destruction) of the bacterial cell, releasing new phage particles that infect additional bacteria. The lytic cycle is characterized by rapid replication and a high rate of bacterial death, making it particularly effective against acute infections. In contrast, some phages follow the lysogenic cycle, during which they integrate their genetic material into the host's genome, becoming a prophage. This integration allows the phage to replicate with the bacterial DNA without causing immediate harm. Under certain conditions, the prophage can switch to the lytic cycle, leading to bacterial cell lysis and the release of new phage particles [12]. Phages exhibit high specificity for their bacterial hosts, often targeting specific strains or species. This specificity is primarily determined by the interaction between phage surface proteins and bacterial receptors [13]. The effectiveness of phage therapy is influenced by several factors, including adsorption (the initial binding of phages to bacteria), which is crucial for infection success, and bacterial resistance mechanisms, such as altering surface receptors or employing restriction-modification systems to evade phage attacks [13]. The interaction between phages and the host immune system is complex. When phages are introduced into the body, they can trigger immune responses, particularly the production of antibodies. These immune responses can have varied effects on phage therapy. The host may produce specific antibodies against phages, leading to the rapid clearance of phages from circulation [14]. The extent of this antibody response depends on factors such as the type of phage, dosage, and route of administration. In some cases, high levels of antibodies may not necessarily correlate with therapeutic outcomes, indicating that the immune response can be both beneficial and detrimental. However, some phages have evolved mechanisms to persist despite host immune responses, such as encapsulation or adaptation to the host environment. This can enhance their ability to evade immune detection and prolong their activity against bacterial targets [14].

Current applications

Phage therapy is increasingly recognized as a viable alternative to traditional antibiotics, with applications spanning various fields and demonstrating significant potential. One of its most notable uses is in treating bacterial infections, especially those involving multidrug-resistant bacteria that are challenging to address with conventional antibiotics [15]. For example, phage therapy has successfully managed chronic infections, such as a case involving a Siamese cat with a surgical wound infected by multidrug-resistant Pseudomonas aeruginosa. The cat was treated with a combination of a specific phage and antibiotics, resulting in complete healing after 14 weeks. This success underscores the potential of phage therapy in treating persistent infections in both humans and animals [15]. Another promising application of phage therapy is in personalized medicine. This approach tailors treatment to the specific bacterial strains infecting individual patients, enhancing therapeutic effectiveness [16]. In veterinary medicine, personalized phage therapy has been used to treat antibiotic-resistant pet infections. For instance, a specific phage was applied topically to successfully address an infection in a dog, demonstrating how customization can effectively target the pathogen responsible for the infection. This personalized approach improves treatment outcomes and reduces the risk of collateral damage to beneficial bacteria [16]. In veterinary medicine, phage therapy is increasingly considered a viable alternative to antibiotics. It has been used to treat various infections in livestock and companion animals [17]. Phage therapy has shown effectiveness in managing conditions such as mastitis in cattle, salmonellosis in poultry, and respiratory diseases in pigs. Studies indicate that phage treatments can significantly reduce bacterial loads and prevent disease, highlighting phage therapy's potential to enhance animal health while decreasing reliance on antibiotics. This shift is particularly relevant given the growing concerns about antibiotic resistance in the veterinary sector [17]. Phage therapy also holds promise in food safety, as it decontaminates and preserves food products. Phages target and eliminate pathogenic bacteria in food, thus improving food safety and extending shelf life [18]. Research has demonstrated that phage treatments can effectively reduce the presence of pathogens such as Salmonella and E. coli in various food items, making phage therapy a valuable tool in food production and safety protocols. Producers can mitigate the risks associated with bacterial contamination by incorporating phage therapy into food safety practices, ultimately benefiting public health [18]. Current applications of phage therapy across these diverse fields are summarized in Table 1.

**Table 1:** Current applications of phage therapy across diverse fields

Application	Targeted Pathogens	Phage Type/Strain	Clinical Status	Use Case	Challenges
Bacterial infections [19]	Escherichia coli, Klebsiella pneumoniae, Pseudomonas aeruginosa	Bacteriophage cocktails	Clinical trials approved in certain countries	Treatment of multidrug-resistant bacterial infections	Emergence of phage resistance and regulatory hurdles
Wound care [20]	Staphylococcus aureus, Pseudomonas aeruginosa	Tailored phage formulations	FDA compassionate use programs	Chronic wound infection management	Standardization of phage dosing
Food safety [21]	Listeria monocytogenes	Bacteriophage P100	Approved for food products (US, EU)	Prevention of bacterial contamination in ready-to-eat foods	Phage survival in food matrices
Veterinary medicine [22]	Escherichia coli, Salmonella	Custom phage preparations	Experimental, used in certain regions	Treatment of livestock infections	Phage-host range limitations
Agriculture [23]	Xanthomonas campestris	Biocontrol phages	Field trials	Prevention of bacterial plant diseases	Environmental stability of phages
Biotechnology & biosensing [24]	E. coli, Bacillus anthracis	Engineered phages	Preclinical development	Detection of bacterial contaminants	Phage stability and sensitivity
Cancer therapy (oncolytic phages) [25]	Various bacterial species	Oncolytic phage strains	Early research, preclinical trials	Phage-mediated bacterial targeting of tumors	Limited research and clinical validation are needed

Innovations and advances

Innovations and advancements in phage therapy are significantly enhancing its efficacy and expanding its applications. One of the most promising areas of development is genomics and synthetic biology approaches. Researchers are engineering bacteriophages to improve their therapeutic effectiveness by manipulating their genomes [26]. This includes enhancing their ability to target specific bacterial strains, increasing their stability, and boosting their resistance to bacterial defenses. Techniques such as CRISPR-Cas9 are being utilized to modify phages, creating phages that can effectively combat antibiotic-resistant bacteria by directly targeting and disrupting bacterial DNA. These advancements are vital for improving treatment outcomes for patients with severe infections [27]. Another key innovation is the creation of phage libraries and the application of phage display technologies. Phage display technology has revolutionized the development of extensive collections of genetically engineered phages that can be screened for their ability to bind to specific bacterial targets. This capability allows researchers to develop broad-spectrum phage therapies by identifying and selecting phages targeting multiple bacterial strains [28]. The ability to create libraries with up to 101010^{10}1010 different variants enables the rapid identification of effective phages for therapeutic use. The biopanning process, involving repeated cycles of selection and amplification, is crucial for enriching phage clones with high binding affinity to targeted pathogens, thus enhancing the therapeutic arsenal against bacterial infections [29]. Combination therapies that integrate phage therapy with traditional antibiotics or other treatment modalities are also emerging as a strategy to overcome antibiotic resistance. This approach leverages the complementary mechanisms of action of phages and antibiotics to enhance overall treatment effectiveness [30]. For instance, while antibiotics may eliminate a significant portion of the bacterial population, phages can target and eradicate the remaining resistant bacteria, reducing the likelihood of relapse and promoting more comprehensive infection eradication. This synergistic effect is being explored in various clinical settings, potentially leading to improved patient outcomes and a more robust approach to managing difficult-to-treat infections [31]. Finally, innovations in novel delivery systems are crucial for the successful application of phage therapy. Researchers are developing new formulations and delivery mechanisms to enhance phage stability, bioavailability, and targeted delivery to infected sites. For example, encapsulation techniques using nanoparticles or hydrogels can protect phages from degradation in the gastrointestinal tract or bloodstream, ensuring they reach their bacterial targets effectively [32]. Additionally, advancements in aerosolized phage delivery systems are being explored for treating respiratory infections, allowing for localized treatment while minimizing systemic exposure. These innovations in delivery methods are essential for maximizing the therapeutic potential of phage therapy and ensuring its successful integration into clinical practice [33]. Innovations and advances in phage therapy are summarized in Table 2.

**Table 2:** Innovations and advances in phage therapy

Innovation/Advance	Description	Key Benefits	Current Status	Challenges
Engineered phages [34]	Genetic modification of phages to enhance specificity, potency, or stability	Targeted bacterial elimination, overcoming bacterial resistance	Preclinical and experimental stages	Ethical concerns, regulatory hurdles
Phage-encoded enzymes [35]	Use of lysins and depolymerases produced by phages to break down bacterial walls	Direct bacterial cell lysis useful against antibiotic-resistant strains	Research and early clinical trials	Stability and delivery challenges
Phage cocktail therapy [36]	Combination of multiple phages to target a broad range of bacterial strains	Prevents bacterial resistance, broadens target range	Approved in some regions, clinical use	Phage interactions, standardization issues
CRISPR-Cas phage engineering [27]	Integration of CRISPR-Cas systems in phages to selectively target bacterial DNA	Precision targeting of bacterial genomes	Research stage	Off-target effects, regulatory approval
Phage encapsulation techniques [37]	Encapsulating phages in polymers or liposomes for controlled release	Improved phage stability and bioavailability	Experimental stages	Scaling and cost-effective production
Bacteriophage-derived nanomaterials [38]	Phage-based nanoparticles for drug delivery and diagnostic purposes	Targeted drug delivery, enhanced diagnostics	Early research, preclinical development	Manufacturing complexity, scalability
Synergistic phage-antibiotic therapy [39]	Combining phages with antibiotics to improve efficacy against infections	Reduced antibiotic doses, enhanced bacterial clearance	Clinical trials, some approved therapies	Interaction between phages and antibiotics
Oncolytic phage therapy [40]	Phages engineered to target cancer cells or tumors via bacterial colonization	Potential for cancer treatment with reduced side effects	Preclinical trials	Lack of human trials, complex tumor environments
Phage-based vaccines [41]	Use of phages as platforms for vaccine development	Cost-effective, scalable, and versatile vaccine production	Research and preclinical studies	Immunogenicity and delivery

Challenges and limitations

The challenges and limitations of phage therapy are complex and span regulatory, developmental, efficacy, and ethical dimensions. Each of these areas presents distinct obstacles that must be addressed to successfully integrate phage therapy into mainstream medical practice [42]. One of the major challenges is regulatory in nature, as phage therapy is often classified alongside traditional antibiotics. Regulatory agencies require phage therapies to undergo rigorous approval processes akin to those for new drugs, which can be cumbersome and time-consuming. This includes demonstrating safety and efficacy through randomized controlled trials, a requirement that has proven difficult given the unique nature of phages as living entities that evolve and adapt in real time [43]. Early clinical studies often lacked adequate controls and employed crude preparations, leading to current safety concerns. Moreover, the immunogenicity of phages poses additional complications, as the body may develop an immune response against them, potentially reducing their effectiveness. Thus, carefully selecting and dosing phages is critical to minimizing adverse reactions while ensuring therapeutic efficacy [44]. The production of phage therapies presents its challenges, particularly regarding the need for high-quality, well-characterized phage preparations. Manufacturing processes must ensure that phages are free from contaminants and antibiotic resistance genes, complicating standardization efforts. Current phage production methods often lack consistency, leading to variability in therapeutic effectiveness [45]. This inconsistency creates a significant barrier to large-scale production and clinical use, as regulatory bodies demand stringent quality controls, which have yet to be universally established in phage therapy. The absence of standardized protocols for phage isolation, characterization, and storage further complicates the development process, making it difficult to produce phage therapies that consistently meet regulatory requirements [45]. Although phages tend to be more specific than antibiotics, the risk of bacteria developing resistance to phage therapy remains a concern. This potential for resistance could undermine the long-term viability of phage treatments [2]. However, the dynamic co-evolution between phages and bacteria offers hope, as phages can adapt and evolve alongside bacterial resistance mechanisms. Strategies such as phage cocktails-combinations of different phages targeting the same bacterial strain-are being explored to mitigate resistance and improve therapeutic outcomes. Continued research is essential to better understand the mechanisms of bacterial resistance to phages and to develop strategies to effectively counteract it [2]. Public perception of phage therapy remains mixed, shaped by historical skepticism and limited awareness of its potential benefits. Many patients and healthcare providers may favor well-established antibiotic treatments over newer, less familiar therapies [46]. Ethical concerns also arise, particularly regarding genetically engineered phages and the implications of personalized medicine, where treatments are tailored to individual patients based on specific bacterial strains. This raises questions about access, equity, and the potential for exploitation in the development of phage therapies. Education and outreach are critical to improving public understanding of phage therapy and addressing ethical concerns surrounding its use [46]. The challenges and limitations of phage therapy are summarized in Table 3.

**Table 3:** Challenges and limitations in phage therapy

Challenge/Limitation	Description	Impact on Phage Therapy	Potential Solutions
Bacterial resistance to phages [2]	Bacteria can develop resistance to specific phages over time	Limits long-term efficacy of phage therapy	Use of phage cocktails, engineering phages to overcome resistance
Phage host range specificity [36]	Phages often target only specific bacterial strains	Limits the broad-spectrum applicability of phage therapy	Development of phage cocktails or broad-spectrum engineered phages
Immune system clearance [47]	The human immune system may clear phages before they act on bacteria	Reduces phage efficacy and therapeutic outcome	Encapsulation of phages or use of immunosuppressants
Regulatory and ethical hurdles [15]	Lack of standardized regulations for phage therapy	Delays in clinical implementation and widespread adoption	Establish clear regulatory guidelines for clinical use
Production and purification challenges [48]	Large-scale production and purification of phages can be complex and costly	Limits the scalability and commercial availability of phage products	Development of cost-effective and scalable production methods
Phage-host interactions [49]	Uncertainty around how phages interact with human cells and microbiota	Potential off-target effects or disturbance of beneficial bacteria	In-depth research on phage-human microbiota interactions
Standardization of phage preparations [42]	Difficulty in standardizing phage doses, strains, and preparations	Inconsistent treatment outcomes across different patients	Development of standardized phage therapy protocols
Phage storage and stability [50]	Phages are sensitive to environmental factors like temperature and pH	Limits their shelf-life and effectiveness	Advanced storage solutions like lyophilization and encapsulation
Limited clinical trials and data [51]	Lack of large-scale randomized controlled trials for phage therapy	Restricts evidence-based adoption in clinical settings	Conducting more large-scale clinical trials
Patient-specific tailoring [2]	Phage therapy often needs to be customized for individual patients	Delays in treatment initiation and complicates treatment logistics	Development of rapid phage matching and diagnostics

Future directions

The future of phage therapy looks promising, with ongoing research and clinical trials poised to overcome current challenges and regulatory advancements likely to facilitate its broader clinical application. Key areas of emerging research include the development of genetically modified phages using CRISPR-Cas technology to enhance efficacy against resistant bacterial strains, the concept of personalized phage therapy tailored to individual patients based on their specific bacterial infections, the creation of broad-spectrum phage cocktails targeting multiple bacterial strains simultaneously, and a deeper understanding of phage-biofilm interactions to improve treatments for chronic infections [52]. The clinical landscape for phage therapy is rapidly advancing, with numerous trials underway. As of March 2023, there are 45 clinical trials listed on clinicaltrials.gov, reflecting a significant increase in recent years and a growing interest in phage therapy as a viable treatment option [15]. Ongoing studies include multi-center trials evaluating the safety and efficacy of phage therapies for various infections, particularly those caused by antibiotic-resistant bacteria. However, a major challenge remains the variability in patient responses to phage therapy due to differences in bacterial strains and host microbiomes. This necessitates carefully selecting and characterizing phages for each clinical application [2]. The regulatory landscape for phage therapy is also evolving, with potential changes to facilitate its use. Regulatory agencies, including the FDA, are becoming more receptive to phage therapy, especially in cases where conventional treatments have failed, including using compassionate protocols for patients with severe infections [53]. There is also a push for clearer guidelines on the design and execution of clinical trials for phage therapy, including the standardization of treatment protocols and data collection methods to enhance the reliability of trial outcomes. Increased funding from governmental and private sources is crucial for advancing phage therapy research, as recent NIH grants aimed at studying phage therapy reflect a growing recognition of its potential as a critical tool in combating antimicrobial resistance [53]. Future directions in phage therapy are detailed in Table 4.

**Table 4:** Future directions in phage therapy

Future Direction	Description	Potential Impact	Current Status
Personalized phage therapy [54]	Tailoring phage treatments based on patient-specific bacterial infections	Increased efficacy and targeted treatments	Early research, clinical trials in progress
Phage-CRISPR systems [55]	Integrating CRISPR-Cas technology with phage therapy to target specific bacterial genes	Precision targeting of multidrug-resistant bacteria	Preclinical research
Phage therapy in combination with probiotics [56]	Using phages alongside probiotics to restore healthy microbiota while eliminating pathogens	Improved gut health and infection control	Experimental studies underway
Phage delivery via nanotechnology [57]	Encapsulating phages in nanoparticles for controlled, targeted delivery	Enhanced stability, targeted delivery to infection sites	Early research stages
Synthetic biology for phage engineering [58]	Designing synthetic phages with enhanced capabilities, such as improved host range or resistance to immune clearance	Customizable and more effective phage therapies	Research and development phase
Integration into mainstream medicine [59]	Collaborating with pharmaceutical companies to integrate phage therapy into standard medical treatments	Broader acceptance and clinical implementation	Ongoing partnerships and regulatory progress
Phage banks for rapid deployment [60]	Establishing large-scale phage banks for rapid matching of phages to bacterial infections	Faster response to emergent antibiotic-resistant infections	Phage banks under development in some regions
Phage therapy for chronic diseases [15]	Investigating the use of phages to treat chronic diseases such as cystic fibrosis or Crohn's disease	Potential new treatment avenues for chronic infections	Early experimental stages
Phage therapy in immunocompromised patients [61]	Developing phage therapies for use in patients with weakened immune systems, such as cancer patients or organ transplant recipients	Safer alternative treatments for vulnerable populations	Preclinical studies
Phage-antibiotic synergism exploration [30]	Further studying the synergistic effects of phages combined with antibiotics to combat resistant infections	Reduced need for high antibiotic doses, minimizing side effects	Ongoing clinical trials
Phage vaccines [62]	Utilizing phages as platforms for delivering vaccine antigens	Cost-effective and scalable vaccine production	Early research and preclinical development

## Conclusions

In conclusion, the evolution of phage therapy represents a promising and potentially transformative advancement in the fight against bacterial infections, especially in the context of rising antibiotic resistance. The historical journey of phage therapy, from its early successes to its decline and recent resurgence, underscores its enduring relevance and potential. As antibiotic resistance continues to challenge conventional treatment options, phage therapy offers a targeted and adaptable approach that can complement existing therapies and address infections that have become resistant to antibiotics. Advances in genetic engineering, synthetic biology, and personalized medicine are enhancing the effectiveness and applicability of phage therapy, making it a valuable tool in modern medicine. This review highlights the importance of continuing research and development in this field and the need to overcome current challenges and regulatory hurdles. By integrating phage therapy into contemporary treatment regimens, we can better address the growing antibiotic resistance crisis and improve outcomes for patients with difficult-to-treat bacterial infections.
